# Hyperacusis is associated with smaller gray matter volumes in the supplementary motor area

**DOI:** 10.1016/j.nicl.2023.103425

**Published:** 2023-04-29

**Authors:** Punitkumar Makani, Elouise A. Koops, Sonja J. Pyott, Pim van Dijk, Marc Thioux

**Affiliations:** a Department of Otorhinolaryngology-Head and Neck Surgery, University of Groningen, University Medical Centre Groningen, P.O. Box 30.001, 9700 RB Groningen, the Netherlands; b Graduate School of Medical Sciences (Research School of Behavioural and Cognitive Neurosciences), University of Groningen, FA30, P.O. Box 196, 9700 AD Groningen, the Netherlands; c Department of Radiology, Massachusetts General Hospital-Harvard Medical School, Boston, USA

**Keywords:** Audition, Hearing loss, Hypersensitivity, Sound-evoked functional MRI, Surface-based morphometry, Tinnitus, Voxel-based morphometry

## Abstract

Hyperacusis is a disorder in loudness perception characterized by increased sensitivity to ordinary environmental sounds and associated with otologic conditions, including hearing loss and tinnitus (the phantom perception of sound) as well as neurologic and neuropsychiatric conditions. Hyperacusis is believed to arise centrally in the brain; however, the underlying causes are unknown. To gain insight into differences in brain morphology associated with hyperacusis, we undertook a retrospective case-control study comparing whole-brain gray matter morphology in participants with sensorineural hearing loss and tinnitus who either scored above or below the threshold for hyperacusis based on a standard questionnaire. We found that participants reporting hyperacusis had smaller gray matter volumes and cortical sheet thicknesses in the right supplementary motor area (SMA), independent of anxiety, depression, tinnitus burden, or sex. In fact, the right SMA volumes extracted from an independently defined volume of interest could accurately classify participants. Finally, in a subset of participants where functional data were also available, we found that individuals with hyperacusis showed increased sound-evoked responses in the right SMA compared to individuals without hyperacusis. Given the role of the SMA in initiating motion, these results suggest that in hyperacusis the SMA is involved in a motor response to sounds.

## Highlights

• Hyperacusis is characterized by hypersensitivity to ordinary environmental sounds.
• Gray matter in the supplementary motor area is reduced in hyperacusis.
• Sound-evoked responses are increased in the same brain region.
• Our results suggest impaired motor responses to sound in hyperacusis.

## 1 Introduction

Hyperacusis is characterized by an increased sensitivity to ordinary environmental sounds of mild to moderate intensity (Anari et al., 1999, Baguley, 2003, Baguley and Hoare, 2018, Nemholt et al., 2015). Environmental sounds that are not uncomfortably loud for most people can be bothersome or even painful for individuals with hyperacusis and trigger avoidance behaviors (Baguley and Hoare, 2018, Fackrell et al., 2019). Hyperacusis is distinguished from misophonia, which involves strong emotional reactions to specific sounds, like someone chewing or whispering (Jastreboff and Jastreboff, 2014, Nemholt et al., 2015). The overall prevalence of hyperacusis in the general population is estimated to be about 17% (Ren et al., 2021). While hyperacusis can occur as a primary complaint, it is frequently comorbid with conditions such as William syndrome and autism (reported prevalence of 95% and 63%, respectively) (Ren et al., 2021) and can occur following acute head trauma, neck injury, and various otologic conditions, many involving stapedial reflex dysfunctions (Baguley, 2003, Baguley and Hoare, 2018, Myne and Kennedy, 2018, Nemholt et al., 2015, Noreña et al., 2018, Potgieter et al., 2020). In the adult population, hyperacusis is strongly associated with hearing loss and tinnitus (Cederroth et al., 2020, Ren et al., 2021). Indeed, up to 90% of individuals with hyperacusis report concurrent tinnitus (Anari et al., 1999), up to 63% of individuals with tinnitus report hyperacusis (Ren et al., 2021), and up to 59% of individuals with hyperacusis have hearing loss (Paulin et al., 2016). In addition, hyperacusis has been associated with anger, anxiety, depression, frustration, insomnia, and stress, resulting in a significant reduction in the quality of life (Baguley, 2003, Baguley and Hoare, 2018, Schecklmann et al., 2014).

Electrophysiological studies in animals with temporary hearing loss induced by salicylate exposure or noise-overexposure suggest that hyperacusis could be the consequence of increased neural gain along the auditory pathway (Auerbach et al., 2019, Chen et al., 2015, Martel and Shore, 2020, Wong et al., 2020). In this model, increased central gain compensates for the loss of peripheral auditory input and results in an enhanced cerebral response to external sounds. In support of this hypothesis, functional neuroimaging studies in humans have found hyperacusis to be associated with enhanced sound-evoked responses in the auditory cortex and the inferior colliculus (Gu et al., 2010, Koops and van Dijk, 2021). Interestingly, although decreased sound tolerance is likely an auditory phenomenon, a previous investigation using resting-state electroencephalography concluded that non-auditory brain regions might also be involved in the pathophysiology of hyperacusis, with the orbitofrontal cortex, the anterior cingulate cortex, and the supplementary motor area showing enhanced beta waves at rest (Song et al., 2014).

In this retrospective case-control study, we examined gray matter differences associated with hyperacusis in a group of adults with sensorineural hearing loss and tinnitus who had taken part in previous neuroimaging studies in our laboratory. We provide convincing evidence that hyperacusis is associated with structural and functional differences in the right supplementary motor area (SMA). These findings provide new insights into the structural and functional mechanisms that might underlie hyperacusis.

## 2 Materials and methods

The data for this study were gathered from two previous neuroimaging studies conducted between 2004 and 2019 at the Department of Otorhinolaryngology of the University Medical Centre Groningen (UMCG) (Boyen et al., 2013, Koops and van Dijk, 2021). Studies were conducted in accordance with the principles of the Declaration of Helsinki and approved by the UMCG medical ethical committee (METc), which also gave approval for the reanalysis of the data (METc number: 2020/347). All participants gave written informed consent. Participants structural MRI data and hearing thresholds were available from both studies, as well as responses to standard questionnaires about anxiety, depression, handedness, hyperacusis, and tinnitus burden (Boyen et al., 2013, Koops and van Dijk, 2021). Sound-evoked functional MRI data from one of the original studies were also re-investigated in a supplementary analysis (Koops & van Dijk, 2021).

### 2.1 Participants

In total, 73 participants with bilateral sensorineural hearing loss and tinnitus were included from the two studies (Boyen et al., 2013, Koops and van Dijk, 2021). Hearing thresholds were assessed with pure tone audiometry at octave frequencies ranging from 0.25 to 8 kHz. Hyperacusis was evaluated with the Hyperacusis Questionnaire (HQ) (Khalfa et al., 2002). In addition, tinnitus burden was measured with the Tinnitus Handicap Inventory (THI) (Newman et al., 1996), the presence of anxiety and depression were assessed with the Hospital Anxiety and Depression Scale (HADS) (Zigmond & Snaith, 1983), and handedness was measured with the Edinburgh Handedness Inventory (EHI) (Oldfield, 1971). Four participants were excluded from the current investigation due to missing data, and three additional participants were excluded as a result of low image quality (*see* section 2.3*. MRI data preprocessing and quality control*). Sixty-six participants were therefore included in the main analyses.

A cut-off score of ≥22 on the 14-item Hyperacusis Questionnaire was used to define the presence or absence of hyperacusis. This cut-off ensures that 95% of the participants classified with hyperacusis have an Uncomfortable Loudness Level below or equal to 77 dB Hearing Level (dB HL) (Aazh & Moore, 2017). Participants who score below 22, on the other hand, can tolerate pure tones of 86 ± 12 dB HL (Aazh & Moore, 2017). With these criteria, 25 (38%) of the 66 participants in our group were classified as having hyperacusis (mean age ± standard deviation = 59.5 ± 7.9 years, 10 females), and 41 participants who scored below the cut-off score were included in the control group (mean age ± standard deviation = 58.3 ± 10.5 years, 9 females).

### 2.2 Brain imaging

For each participant, high-resolution 3-dimensional T1-weighted anatomical images were obtained using a 3-Tesla Philips Intera scanner (*Philips Medical System, Best, The Netherlands*) equipped with a phase-array head coil (SENSE) with either 8-channel (Boyen et al., 2013) or 32-channel (Koops & van Dijk, 2021). Fast-field echo images were acquired with the following parameters for the two studies (Boyen et al., 2013*/*Koops & van Dijk, 2021): repetition time = 9/10.4 ms, echo time = 3.5/5.7 ms, field of view = 232 × 256/256 × 224 mm, flip angle = 8˚, number of slices = 170/160, slice thickness = 1 mm, slice gap = 0 mm, scan time = 251/614 sec, reconstructed voxel size = 1 × 1 × 1 mm.

In a supplementary analysis, sound-evoked functional MRI data from one of the original studies were also re-investigated and included (Koops & van Dijk, 2021). In this study, the cerebral response to pure tones of various frequencies (0.25 to 8 kHz, loudness matched to a 1 kHz tone at 40 dB SPL) was recorded with echo-planar imaging in a sparse-sampling experiment (repetition time = 10 sec, acquisition time = 2 sec, stimulus duration = 7.5 sec, reconstructed voxel size = 2 × 2 × 2 mm, full brain coverage). Activation evoked by the pure tones was contrasted with a silence condition. During both the tone and silence conditions, participants had to perform a concurrent visual valence task, which involved a motor response on a keypad. Statistical parametric maps of the contrasts between sound and silence were retrieved from this study for each available participant and used to compare the sound-evoked cerebral response between two groups under investigation here.

### 2.3 MRI data preprocessing and quality control

T1-weighted anatomical images were preprocessed using CAT12.7 (*v.1653, Structural Brain Mapping Group, University of Jena, Germany,* *http://dbm.neuro.uni-jena.de/cat12/*) and SPM12 (*v.7487, Wellcome Centre for Human Neuroimaging, University College London,* *https://www.fil.ion.ucl.ac.uk/spm/*), in MATLAB R2020a (*The MathWorks, Natick, MA, USA*). Prior to preprocessing, REC/PAR Philips images were converted to NIFTI (*Neuroimaging Informatics Technology Initiative*) format using dcm2niix (*v.1.2.20200331,* *https://github.com/rordenlab/dcm2niix/*). Preprocessing steps followed the recommendations of CAT12.7. Briefly, T1-weighted anatomical images were initially corrected for bias, noise, and global intensity, and then segmented into gray matter, white matter, and cerebrospinal fluid (CSF) images. Subsequently, gray matter images were normalized to the Montreal Neurological Institute (MNI152) template using the Diffeomorphic Anatomical Registration Through Exponentiated Lie algebra (DARTEL) algorithm, using affine followed by non-linear registration (Ashburner, 2007). Normalized gray matter images were resampled to 1.5 × 1.5 × 1.5 mm voxels using a nearest neighbour interpolation. To account for volume changes induced by normalization, the normalized gray matter images were modulated by the resulting Jacobian determinant (Ashburner, 2007).

The quality of the normalized modulated gray matter images was assessed in two steps. First, gray matter images were visually inspected to detect potential segmentation issues (e.g., tissue misclassification). Second, gray matter image homogeneity was assessed in CAT12.7 based on the weighted overall image quality index, and the average voxel-wise correlation between images. This second step led to the removal of three datasets with a Mahalanobis distance larger than 1.5 IQR compared to the rest of the data. Finally, the normalized modulated gray matter images were smoothed using a Gaussian smoothing kernel of 12 mm full-width half-maximum (FWHM).

### 2.4 Statistical analyses

#### 2.4.1 Demographic, audiometric, and questionnaires data

Statistical analyses of demographic, audiometric, and questionnaires data were performed using SPSS (*v.27, IBM Corp., Armonk, New York, USA*). For the categorical variable sex, a Chi-square test was used to test whether groups differed in sex distribution. Based on the outcome of the Shapiro-Wilk normality test, continuous variables were tested for between-group comparisons using either independent samples Welch’s t-tests or independent samples Mann-Whitney U tests. Lastly, Spearman’s Rank correlation coefficients were computed to assess the strength and direction of relationships between pairs of questionnaire scores. A statistical threshold of P ≤ 0.05 was used for all analyses.

#### 2.4.2 Whole-brain gray matter volumes analyses

Between-group differences in gray matter volumes were investigated voxel-wise with a General Linear Model (GLM). Two whole-brain two-sample *t*-test analyses were conducted with different sets of covariates to test for the effect of possible confounds. The total intracranial volume (TIV; computed as the sum of the gray, white, and CSF compartments) was entered as a covariate in the model in both analyses to correct for differences in brain sizes (Pell et al., 2008). In the first analysis, TIV was the only covariate. In the second analysis, age, handedness scores, and hearing thresholds were entered into the model with TIV. Hearing thresholds were calculated as the pure tone average (PTA) in dB HL across all tested frequencies (0.25, 0.5, 1, 2, 4, and 8 kHz) for both ears. A statistical threshold of P ≤ 0.05 family-wise error (FWE) corrected at the voxel-level was used for both analyses. Although very strict, this statistical threshold is recommended for voxel-based morphometry (VBM) analyses (Ridgway et al., 2008). It greatly reduces the reporting of false positive findings and allows us drawing conclusions at the voxel by opposition to cluster level.

#### 2.4.3 Volume-of-interest analyses in the right SMA

Considering the primary results, several supplementary analyses were conducted in a volume-of-interest (VOI) restricted to the right supplementary motor area (SMA). The right SMA VOI was obtained from a published *meta*-analysis of functional MRI data, which used both task-related activation coordinates and anatomical boundaries to delineate motor cortex areas (*freely available as the Human Motor Area Template (HMAT),* *http://lrnlab.org/*) (Mayka et al., 2006). Gray matter volumes were extracted in this right SMA VOI for each participant using get_totals MATLAB script (*freely available from* *http://www0.cs.ucl.ac.uk/staff/g.ridgway/vbm/get_totals.m/*) and further analysed in SPSS.

##### 2.4.3.1 Effect of anxiety, depression, tinnitus burden

To assess the possible impact of anxiety, depression, or tinnitus burden on the results, Spearman’s Rank correlation coefficients between the right SMA gray matter volumes (HMAT VOI) and either the HADS or the THI scores were computed across all participants using a statistical threshold of P ≤ 0.05.

##### 2.4.3.2 Effect of sex

2 × 2 between-groups ANOVA was conducted to examine the possible interaction between the effect of sex and hyperacusis on the right SMA gray matter volumes (HMAT VOI) using a statistical threshold of P ≤ 0.05.

##### 2.4.3.3 Receiver operating characteristic curve

The receiver operating characteristic (ROC) curve was obtained from the data to evaluate the possibility of classifying participants with hyperacusis based on their right SMA gray matter volumes (HMAT VOI). The ROC analysis was conducted in SPSS using logistic regressions.

##### 2.4.3.4 Cortical thickness

Cortical thickness was estimated in CAT12.7 by surface-based morphometry using topological correction and spherical mapping (Yotter et al., 2011a, Yotter et al., 2011b) Cortical thickness VOI of the right SMA was extracted from the Human Connectome Project Multi-Model Parcellation (HCP-MMP1) Atlas (Sheets et al., 2021) and compared between groups with independent samples Welch’s *t*-test using a statistical threshold of P ≤ 0.05.

##### 2.4.3.5 Sound-evoked activity

The Blood Oxygen Level Dependent (BOLD) response to sounds was compared between participants with and without hyperacusis. The analysis was conducted within the right SMA (HMAT VOI). The average BOLD response to sounds was compared between groups using SPM12 (P ≤ 0.05 FWE corrected at the voxel level), corrected for age and sex. statistical threshold of P ≤ 0.05 was used for all analyses.

## 3 Results

### 3.1 Group characteristics

**Fig. 1:**
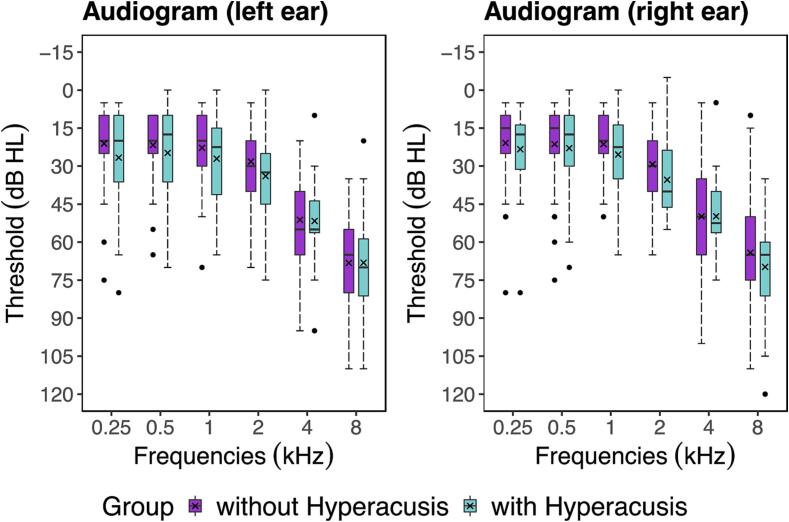
Audiometric assessments for the groups with and without hyperacusis. All participants had sensorineural hearing loss and tinnitus. There was no group difference in hearing thresholds (dB HL) at any of the tested frequencies.

The groups of participants with and without hyperacusis did not differ significantly in terms of age (U = 507, Z = –0.1, P = 0.942), sex distribution [χ^2^(1) = 2.5, P = 0.116], total intracranial volume [t(62.9) = –1.5, P = 0.135], or weighted overall image quality (U = 392.5, Z = –1.6, P = 0.113). Pure tone hearing thresholds were well matched between groups across all tested frequencies (Fig. 1), and the pure tone averages (PTA 0.25–8 kHz, PTA 0.25–1 kHz, and PTA 2–8 kHz) for both ears did not differ between groups [PTA 0.25–8 kHz: U = 401.5, Z = –1.2, P = 0.218; PTA 0.25–1 kHz: U = 449.5, Z = –0.6, P = 0.563; PTA 2–8 kHz: t(46.2) = 0.9, P = 0.381]. However, compared to controls, participants with hyperacusis scored significantly higher on measures of depression (HADS depression: U = 303, Z = –2.7, P = 0.007) as well as tinnitus burden (THI: U = 280.5, Z = –2.8, P = 0.006). There was also a trend towards higher scores on measures of anxiety (HADS anxiety: U = 365, Z = –1.8, P = 0.067) in participants with hyperacusis compared to controls. Demographic, audiometric, and questionnaires data for both groups are summarized in Table 1 (more details: Supplementary Table S1).

**Table 1:** Summarized demographic, audiometric, and clinical data for the groups with and without hyperacusis.

	Group without Hyperacusis	Group with Hyperacusis
	Demographic	
*n*	41	25
Age (years)	58.3 ± 10.5 (27–76)	59.5 ± 7.9 (41–73)
Sex (male/female)	(32/9)	(15/10)

	Audiometric (PTA 0.25–8 kHz for both ears)	
Hearing levels dB HL	35 ± 8.6	38.2 ± 12.3

	Questionaries	
HQ Hyperacusis	13.2 ± 5.2 (0–21)	26.3 ± 3.7 (22–33)*
HADS Anxiety	3.7 ± 3 (0–11)	5.9 ± 4.6 (0–16)
HADS Depression	3.3 ± 3.1 (0–10)	6.2 ± 4.7 (0–16)*
THI Tinnitus burden	27.4 ± 19.4 (4–80)	41.8 ± 20.9 (6–82)*

Mean ± Standard Deviation (Range). *P ≤ 0.05.

### 3.2 Whole-brain VBM reveals smaller right SMA gray matter volumes in participants with hyperacusis

**Fig. 2:**
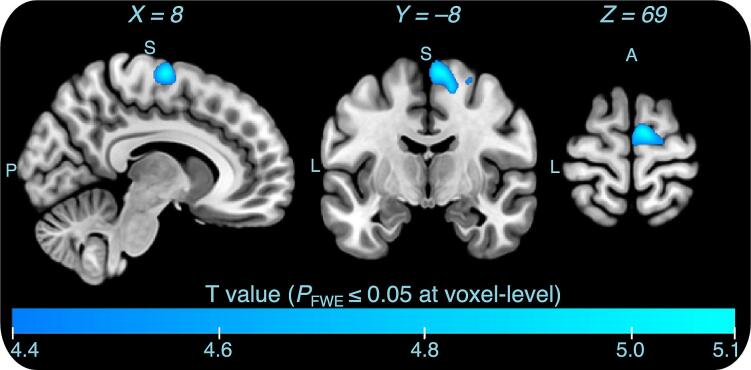
Results of the whole-brain between groups comparison showing smaller gray matter volumes in the right supplementary motor area (SMA) of participants with hyperacusis compared to those without hyperacusis. The analysis accounted for age, handedness score, hearing thresholds (PTA 0.25–8 kHz for both ears), and total intracranial volume (TIV) as confounding variables. One single cluster (265 voxels) of gray matter volumes difference was found in the entire brain, revealing smaller gray matter volumes in the right SMA of participants with hyperacusis, shown in cold color map (peak MNI X, Y, Z - coordinates = 8, –8, 69, T = 5.1, P_FWE_ = 0.016 at the voxel level).

Gray matter volume differences between groups were assessed voxel-wise across the whole brain in two analyses controlling for different sets of potential confounds (P_FWE_ ≤ 0.05 at the voxel level). In the first analysis, the total intracranial volume (TIV) was the only covariate. This analysis revealed a single cluster (244 voxels) of smaller gray matter volumes in the right supplementary motor area of participants with hyperacusis (Supplementary Figure S1, Supplementary Table S2). In the second analysis, age, handedness scores, and hearing thresholds (PTA 0.25–8 kHz for both ears) were added as covariates in the model together with TIV. This analysis returned very similar results, identifying a single cluster (265 voxels) of smaller gray matter volumes in the right supplementary area of participants with hyperacusis (Fig. 2, Table 2). No any brain region showed a significant increase in gray matter volumes associated with hyperacusis. In addition, we re-ran the analysis using the less conservative threshold free cluster enhancement technique (TFCE with FWE correction P ≤ 0.05 using peak-cluster-level). We found a similar albeit larger cluster (14,276 voxels), showing smaller gray matter volumes in participants with hyperacusis compared to those without hyperacusis (Supplementary Figure S2, Supplementary Table S3). This larger cluster encompassed the SMA, pre-supplementary motor area (Pre-SMA), and precentral gyrus (PrC) bilaterally. However, we did not observe any other significant gray matter differences between two groups with and without hyperacusis. Furthermore, we observed that our finding was replicated when treating hyperacusis as a continuous rather than a categorical variable and that the negative correlation between the right SMA gray matter volumes (HMAT VOI) and the hyperacusis scores was largely driven by the attentional subscale of the Hyperacusis Questionnaire (Supplementary Table S4, Supplementary Table S5).

**Table 2:** Results of whole-brain two-sample *t*-test analysis between the groups with and without hyperacusis including age, handedness score, hearing thresholds (PTA 0.25–8 kHz for both ears), and total intracranial volume (TIV) as covariates (P_FWE_ ≤ 0.05 at the voxel-level, height threshold T = 4.4).

Area	P_FWE_ voxel-level	P_FWE_ cluster-level	MNI coordinates			Cluster size k	T-value	Z-value
			X	Y	Z			
	**Group without Hyperacusis > Group with Hyperacusis**							
SMA (right)	0.016	0.004	8	–8	69	265	5.1	4.6
	0.027		15	–6	58		4.5	4.5
	0.038		21	–15	64		4.4	4.4

	**Group without Hyperacusis < Group with Hyperacusis**							
NS	–	–	–	–	–	–	–	–

NS not significant.

### 3.3 Anxiety, depression, or tinnitus burden do not drive smaller gray matter volumes in hyperacusis

The average scores on scales evaluating depression and tinnitus burden differed significantly between participants with and without hyperacusis (Table 1). However, we found no significant correlation between the right SMA gray matter volumes (HMAT VOI) and either the HADS anxiety [r_s_(63) = –0.04, P = 0.765], the HADS depression [r_s_(63) = –0.06, P = 0.614], or the THI tinnitus burden [r_s_(62) = –0.07, P = 0.607] scores. These variables, therefore, do not appear to drive the gray matter volumes difference observed in the right SMA between participants with and without hyperacusis.

### 3.4 No interaction between effect of sex and hyperacusis

Since a previous *meta*-analysis reported differences in the gray matter volumes of the SMA between males and females (Lotze et al., 2019), effect of sex could, therefore, potentially interact with effect of hyperacusis. However, 2 × 2 between-groups ANOVA on the right SMA gray matter volumes (HMAT VOI) revealed a significant effect of hyperacusis [F(1,62) = 18.4, P < 0.001], a trend towards an effect of sex [F(1,62) = 3.6, P = 0.064], but no significant interaction between effect of sex and hyperacusis [F(1,62) = 0.2, P = 0.641].

### 3.5 Right SMA gray matter volumes can accurately classify participants with hyperacusis

**Fig. 3:**
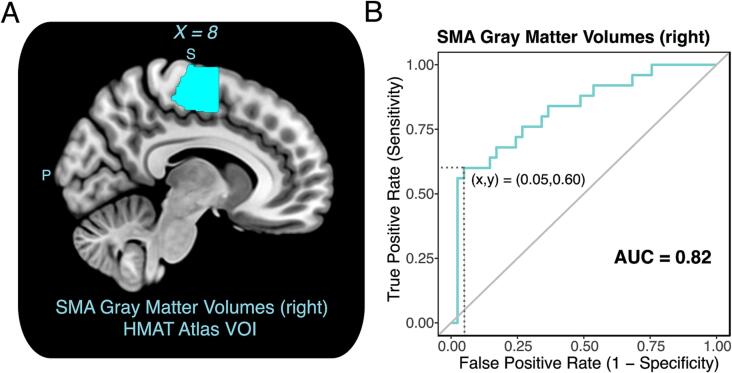
A receiver operating characteristic (ROC) analysis demonstrated the capability to classify patients with hyperacusis based on the gray matter volumes in an independently defined right SMA volume-of-interest. **A:** The right SMA volume-of-interest (VOI in cyan color) was defined according to the Human Motor Area Template (HMAT) atlas, derived from anatomical and functional landmarks. **B:** ROC curve for the detection of participants with hyperacusis based on right SMA gray matter volumes; a classifier was able to distinguish participants with or without hyperacusis with an area under the curve of 0.82 (P < 0.001, 95 %CI 0.72–0.93). About 60% of participants with hyperacusis could be classified based on their right SMA gray matter volumes with<5% false positives. (For interpretation of the references to color in this figure legend, the reader is referred to the web version of this article.)

The receiver operating characteristic (ROC) curve was computed to verify that participants with hyperacusis could reliably be classified based on their right SMA gray matter volumes (HMAT VOI). The area under the curve (AUC) was 0.82 ± 0.1 (P < 0.001), 95% CI = (0.7, 0.9), demonstrating a very good classification performance (Fig. 3). Almost 60% of participants with hyperacusis (identified by their questionnaire scores) could be classified with <5% false positives based on their right SMA gray matter volumes.

### 3.6 Thinner right SMA cortical sheet in participants with hyperacusis

**Fig. 4:**
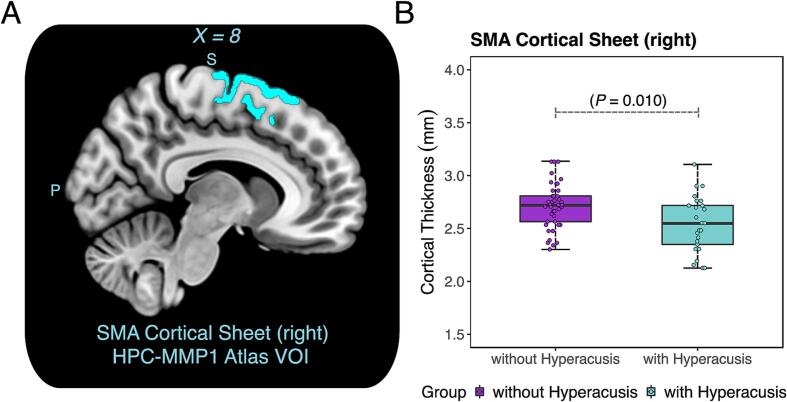
Thinner right supplementary motor area (SMA) cortical sheet in individuals with hyperacusis. **A:** A sagittal view of the right SMA volume-of-interest (VOI in cyan color) of cortical sheet according to the Human Connectome Project Multi-Model Parcellation (HPC-MMP1) Atlas. **B:** The surface-based VOI analysis revealed that the right SMA cortical sheet was thinner in participants with hyperacusis relative to those without hyperacusis [t(42.9) = –2.7, P = 0.010]. (For interpretation of the references to color in this figure legend, the reader is referred to the web version of this article.)

To test whether differences in the right SMA cortical sheet thickness could explain the smaller cortical volumes in this area, thickness values in the right SMA (HPC-MMP1 VOI) were compared between groups. In addition to smaller cortical volumes, the analysis revealed that the right SMA cortical sheet was thinner in individuals with hyperacusis compared to controls [t(42.9) = –2.7, P = 0.010, 95% CI = (–0.3, –0.04)] (Fig. 4). Therefore, it appears that differences in gray matter volumes associated with hyperacusis are at least partly driven by differences in cortical thickness.

### 3.7 Sound-evoked activity in the right SMA is elevated in participants with hyperacusis

**Fig. 5:**
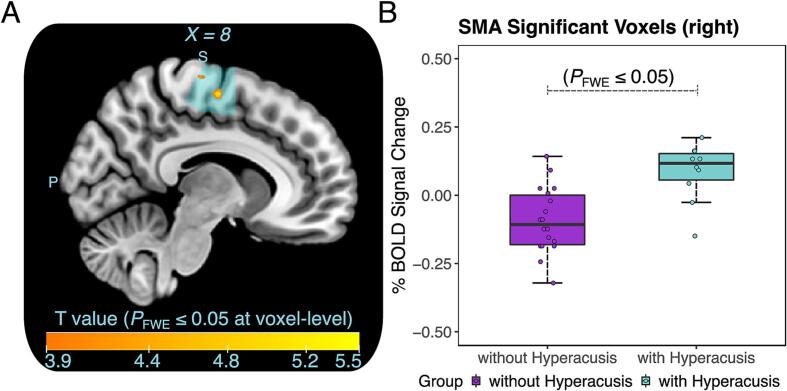
Higher sound-evoked activity in the right supplementary motor area (SMA) of participants with hyperacusis compared to those without hyperacusis. **A:** The analysis conducted in the right SMA volume-of-interest (VOI in cyan colour, identical to the VOI in Fig. 3) showed increased BOLD response to sounds versus baseline in participants with hyperacusis compared to those without hyperacusis (shown in warm color map: cluster size = 30 voxels, peak MNI *X*,*Y*, *Z* – coordinate = 8, –10, 60, T = 5.5, P_FWE_ < 0.001 at the voxel-level). The analysis accounted for age and sex as confounding variables. **B:** Box plots of the average % BOLD signal change in the right SMA voxels showing significantly higher response to 40 dB HL pure tones in participants with hyperacusis. (For interpretation of the references to color in this figure legend, the reader is referred to the web version of this article.)

In a final analysis, we tested the hypothesis that the right SMA would show increased responsiveness to sounds in individuals with hyperacusis, which would suggest a link between sound-evoked cerebral activity and decreased gray matter volumes and thickness in the right SMA. Sound-evoked functional MRI data were available from a subset of 10 participants with hyperacusis (mean age ± standard deviation = 58.8 ± 9.1 years, 1 female) and 18 controls (mean age ± standard deviation = 58.9 ± 10.9 years, 3 females). The two groups were well-matched in terms of age [U = 83.5, Z = –0.3, P = 0.755], sex distribution [χ^2^(1) = 0.2, P = 0.629], and hearing thresholds [PTA 0.25–8 kHz: t(16.2) = –0.5, P = 0.645]. The cerebral activity in the right SMA (HMAT VOI) in response to the presentation of pure tones relative to the activity during a silence condition was compared between the two groups of participants. We found a higher BOLD response (P_FWE_ ≤ 0.05 at the voxel level) in the right SMA of participants with hyperacusis relative to controls, corrected for age and sex (Fig. 5).

## 4 Discussion

The aim of this retrospective study was to investigate potential differences in gray matter associated with hyperacusis. Sixty-six participants with sensorineural hearing loss and tinnitus were included in the final analysis, of whom twenty-five (38%) met the criteria for hyperacusis (Aazh and Moore, 2017, Khalfa et al., 2002). The two groups were well-matched in terms of age, sex distribution, and hearing thresholds. We found that individuals with hyperacusis had smaller gray matter volumes in the right supplementary motor area (SMA) even when considering the potentially confounding effects of age, handedness scores, and hearing thresholds. We found no significant effect of anxiety, depression, or tinnitus burden, and no significant interaction between sex and hyperacusis on the right SMA gray matter volumes. In fact, we found that the right SMA gray matter volumes could accurately classify participants with hyperacusis versus controls with an 82% area under the curve. Moreover, we found that, in the hyperacusis group, the right SMA cortical sheet was thinner, which can partially explain smaller gray matter volumes in this group.

In light of the above results, we predicted that hyperacusis would be associated with enhanced sound-evoked responses in the right SMA. This pattern of larger stimulus-evoked activity and smaller gray matter volumes has been reported for instance in war veterans who developed posttraumatic stress disorder (PTSD), showing reduced gray matter volumes in the amygdala (Morey et al., 2012, O’Doherty et al., 2015) along with enhanced amygdala BOLD response to fearful stimuli (Chiba et al., 2021). According to expectations, we found increased BOLD responses to sounds in the right SMA of participants with hyperacusis compared to controls. The result of this complimentary analysis suggests a link between the right SMA gray matter volumes and hyper-responsiveness of this region to sound stimuli. Although we included sex as a covariate, generalizing findings to both sexes should be done cautiously given the limited number of female participants in this analysis. Future studies should aim to include larger and more sex-balanced groups.

The discovery of hyperacusis-related differences in the right SMA gray matter morphology is, at first, perhaps surprising: the SMA is remote from the auditory system and is known to be involved in action preparation (Nachev et al., 2008). However, previous work indicates that the SMA, in fact, contributes to auditory processing and auditory imagery, and a review concluded that the supplementary motor and pre-motor areas play a role in facilitating motor responses to sounds (Lima et al., 2016). Accordingly, electrophysiological recordings in monkeys showed that some SMA neurons fire only when a movement is preceded by a specific sensory cue, for instance, an auditory stimulus but not a visual or tactile stimulus (Tanji and Kurata, 1982, Tanji and Kurata, 1985). These neurons do not respond to the sole presentation of the sensory cue but rather respond only if the cue is followed by appropriate action. These findings suggest that, in hyperacusis, sounds of moderate intensity might trigger motor programs that correspond to an aversive reaction to environmental sounds. Interestingly, Fried et al. (Fried et al., 1991) reported a left–right specialization of the SMA whereby bilateral movements were elicited exclusively by stimulation of the non-dominant (right) hemisphere. This finding could potentially explain why in the current study hyperacusis was associated with lower gray matter volumes in the right SMA, as the most natural reaction to perceiving a very loud noise is probably to cover one’s ears.

Although the current investigation cannot confirm a causal link between hyperacusis and smaller SMA gray matter volumes and cortical thickness, our findings suggest testable hypotheses. One hypothesis could be that, in participants with hyperacusis, smaller gray matter volumes and cortical thickness and yet enhanced sound-evoked activity in the right SMA reflect the tendency to initiate movements in response to sounds perceived as too loud. In support of this hypothesis, Auerbach et al. (Auerbach et al., 2019) reported that inducing hearing loss and hyperacusis in rats through salicylate injection resulted in faster response latencies to sounds. The effect was dependent on sound intensity and was correlated with an increased sound-evoked neuronal response in the auditory cortex and inferior colliculus at an individual level (Auerbach et al., 2019). These findings, together with our results, suggest that the initiation of a motor reaction to sound stimuli may be facilitated in participants with hyperacusis as a result of peripheral damage and increased central gain along the auditory pathway. Given that some SMA neurons are modality specific, examining whether participants with hyperacusis have shorter reaction times specifically to auditory as opposed to visual or tactile stimuli would suggest a more direct link between increased central gain in the auditory pathway and gray matter alterations in the SMA. Furthermore, differentiating between different types of behavioural reactions to hyperacusis might increase our understanding of the causality between hyperacusis and the SMA gray matter differences. Recent studies in PTSD, for instance, have shown that gray matter changes are dependent on the type of trauma (Meng et al., 2016) as well as the type of behavioural strategy deployed (or not deployed) to avoid trauma (Crombie et al., 2021). Such an investigation would, however, require a more complete assessment of individuals with hyperacusis than afforded by the instruments available in this study (Baguley and Hoare, 2018, Enzler et al., 2021, Fackrell et al., 2015).

An alternative hypothesis could be that hyperacusis results from reduced SMA gray matter volumes and thickness. In this scenario, reduced SMA gray matter volumes would allow hyperacusis to develop following sensorineural hearing loss. This hypothesis is consistent with the observation that, in general, hyperacusis is slightly more prominent in females (Jacquemin et al., 2022), who on average have smaller SMA gray matter volumes than males (Ruigrok et al., 2014). However, the current study found that the effect of hyperacusis on the right SMA gray matter volumes was much larger than the effect of sex and that there was no interaction between sex and hyperacusis. Ultimately, longitudinal studies will be necessary to provide insight into the complex relationship between peripheral damage, hyperacusis susceptibility, and behavioural adaptation/reactivity.

In conclusion, hyperacusis in individuals with sensorineural hearing loss and tinnitus appears to be associated with smaller gray matter volumes and reduced cortical thickness of the right supplementary motor area (SMA). This neuroanatomical difference is robust enough to be noticeable in the relatively small number of participants investigated in this study and, in fact, can successfully classify participants with hyperacusis. Our finding of increased evoked (BOLD) responses to sound stimuli in this area supports the hypothesis that increased central gain associated with hyperacusis occurs not only along the auditory pathway but also in the non-auditory SMA and could lead to changes in SMA gray matter volume and thickness. Further research investigating the link between hyperacusis and the structure and function of the SMA are warranted, not only in individuals with sensorineural hearing loss and tinnitus but also in children who develop hyperacusis following repeated otitis media episodes and individuals with conditions such as Williams syndrome and autism.

## 5 Data availability

The unthresholded statistical parametric map supporting the main finding of this study is openly available at neurovault.org (*https://identifiers.org/neurovault.collection:13313*).

## Funding

This research was supported by the European Research Council (ERC) under the European Union’s Horizon 2020 Research and Innovation Programme (Grant agreement number 764604, TINACT), the Heinsius Houbolt Foundation, the Netherlands Organization for Health Research and Development (ZonMW), the American Tinnitus Association under the Innovative Grants Research Program 2021 (Grant agreement number 91218033), and the Dorhout Mees Family Foundation.

## CRediT authorship contribution statement

**Punitkumar Makani:** Conceptualization, Data curation, Formal analysis, Methodology, Software, Visualization, Writing – original draft. **Elouise A. Koops:** Formal analysis, Investigation, Methodology, Software, Writing – review & editing. **Sonja J. Pyott:** Conceptualization, Funding acquisition, Project administration, Supervision, Writing – review & editing. **Pim van Dijk:** Conceptualization, Funding acquisition, Project administration, Supervision, Writing – review & editing. **Marc Thioux:** Conceptualization, Methodology, Validation, Writing – review & editing.

## Declaration of Competing Interest

The authors declare that they have no known competing financial interests or personal relationships that could have appeared to influence the work reported in this paper.

## Data Availability

The unthresholded statistical parametric map supporting the main finding of this study is openly available at neurovault.org (https://identifiers.org/neurovault.collection:13313).
