# Supplementary material for: Hyperacusis is associated with smaller gray matter volumes in the supplementary motor area
Source: Neuroimage Clin. 2023 Apr 29;38:103425. doi: 10.1016/j.nicl.2023.103425 (PMC10176058; doi:10.1016/j.nicl.2023.103425)
Supplement: Supplementary data 1 [file mmc1.pdf]

Hyperacusis is associated with smaller gray matter volumes in the supplementary motor area

Punitkumar Makani[1,2*], Elouise A Koops[1,3], Sonja J Pyott[1,2], Pim van Dijk[1,2†], Marc Thioux[1,2†]

[†]These authors contributed equally to this work

[1]Department of Otorhinolaryngology-Head and Neck Surgery, University of Groningen, University Medical Centre Groningen, P.O. Box 30.001, 9700 RB Groningen, The Netherlands

[2]Graduate School of Medical Sciences (Research School of Behavioural and Cognitive Neurosciences), University of Groningen, FA30, P.O. Box 196, 9700 AD Groningen, The Netherlands

[3]Department of Radiology, Massachusetts General Hospital-Harvard Medical School, Boston, USA

[*]For correspondence

p.makani@umcg.nl (P.M.)

Competing interests

The authors report no competing interests.

Supplementary Table S1. Overview of demographic, audiometric, questionaries, and morphometric data for the groups with and without hyperacusis.

	Group without Hyperacusis	Group with Hyperacusis	Statistics
	**Demographic**		
n	41	25	-
Age (years)	58.3 ± 10.5 (27 - 76)	59.5 ± 7.9 (41 - 73)	*U* = 507, *Z* = –0.1, *P* = 0.942
Sex (male / female)	(32 / 9)	(15 / 10)	$X^2(1)$ = 2.5, *P* = 0.116
Handedness (L / R / NP / NA)	(5 / 29 / 0 / 7)	(0 / 23 / 2 / 0)	-
	**Audiometric (for both ears)**		
Hearing levels dB HL (PTA 0.25 - 8 kHz)	35 ± 8.6 (20.4 - 57.9)	38.2 ± 12.3 (11.2 - 66.3)	*U* = 401.5, *Z* = –1.2, *P* = 0.218
Hearing levels dB HL (PTA 0.25 - 1 kHz)	21.5 ± 12.4 (7.5 - 68.3)	25 ± 16.6 (5 - 71.7)	*U* = 449.5, *Z* = –0.6, *P* = 0.563
Hearing levels dB HL (PTA 2 - 8 kHz)	48.5 ± 12.6 (28.3 - 15.8)	51.5 ± 13.3 (17.5 - 88.3)	*t*(46.2) = 0.9, *P* = 0.381
	**Questionaries**		
HQ Hyperacusis	13.2 ± 5.2 (0 - 21)	26.3 ± 3.7 (22 - 33)	***t*(62.6) = 11.9, *P* < 0.001**
HQ-attentional	3.7 ± 1.7	7.3 ± 1.4	***U* = 52.2, *Z* = –5.7, *P* < 0.001**
HQ-social	5.4 ± 3.4	10.5 ± 2.4	***t*(56.9) = 6.8, *P* < 0.001**
HQ-emotional	4.1 ± 1.9	8.4 ± 2.1	***t*(46.6) = 7.9, *P* < 0.001**
HADS Anxiety	3.7 ± 3 (0 - 11)	5.9 ± 4.6 (0 - 16)	*U* = 365, *Z* = –1.8, *P* = 0.067
HADS Depression	3.3 ± 3.1 (0 - 10)	6.2 ± 4.7 (0 - 16)	***U* = 303, *Z* = –2.7, *P* = 0.007**
THI Tinnitus burden	27.4 ± 19.4 (4 - 80)	41.8 ± 20.9 (6 - 82)	***U* = 280.5, *Z* = –2.8, *P* = 0.006**
THI-functional	15.6 ± 10.2	25.6 ± 11.8	***U* = 255.5, *Z* = –3.1, *P* = 0.002**
THI-emotional	6.7 ± 6.8	9.6 ± 6.9	*U* = 345.5, *Z* = –1.9, *P* = 0.060
THI-catastrophic	5.2 ± 4.1	6.7 ± 3.9	*U* = 365.5, *Z* = –1.8, *P* =0.081
	**Morphometric**		
TIV (cm^3)	1494.9 ± 139.1	1451 ± 96.3	*t*(62.9) = –1.5, *P* = 0.135
WOIQ (%)	83 ± 3.7	80.9 ± 4.2	*U* = 392.5, *Z* = –1.6, *P* = 0.113

Mean ± Standard Deviation (Range). NA not available; NP no preference; L left; R right.

Supplementary Table S2. Results of the whole-brain two-sample t-test analysis between the groups with and without hyperacusis including total intracranial volume (TIV) as a covariate ($P_{FWE} \leq 0.05$ at the voxel-level, height threshold T = 4.7).

**Area**	***P*FWE voxel-level**	***P*FWE cluster-level**	**MNI coordinates *X***	***Y***	***Z***	**Cluster size *k***	**T-value**	**Z-value**
	**Group without Hyperacusis > Group with Hyperacusis**							
SMA (right)	0.012	0.005	9	–8	69	244	5.2	4.7
	0.049	0.047	20	–15	64	1	4.7	4.3
	**Group without Hyperacusis < Group with Hyperacusis**							
NS	-	-	-	-	-	-		-

NS not significant.

Supplementary Table S3. Results of whole-brain two-sample t-test analysis between the groups with and without hyperacusis including age, handedness score, hearing thresholds (PTA 0.25 - 8 kHz for both ears), and total intracranial volume (TIV) as covariates (TFCE $P_{FWE} \leq 0.05$ at the peak-cluster-level).

Area	TFCE P_{FWE} peak-cluster-level	MNI coordinates *X*	*Y*	*Z*	Cluster size *k*	TFCE-value	
	**Group without Hyperacusis > Group with Hyperacusis**						
SMA	0.001	8	–8	69	14276	3016.2	
Pre-SMA		15	–6	58		2987.8	
PrC (bilateral)		–18	–33	74		2949.3	
	**Group without Hyperacusis < Group with Hyperacusis**						
NS	-	-	-	-	-	-	-

NS not significant. PrC precentral gyrus; Pre-SMA pre-supplementary motor area.

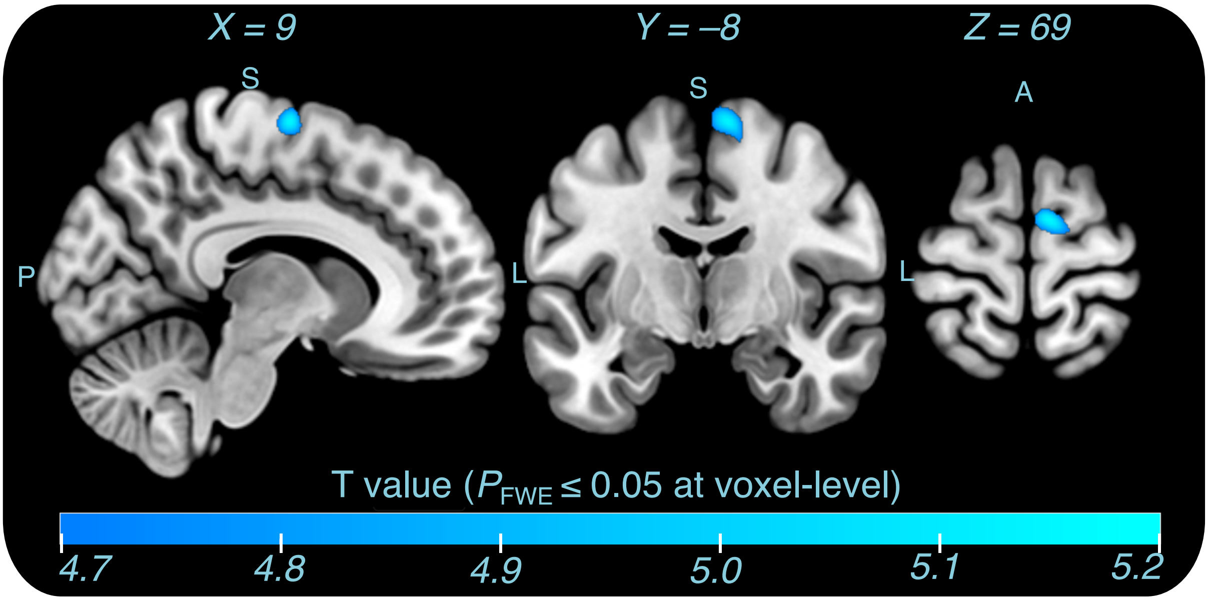

Supplementary Figure S1. Results of the whole-brain between groups comparison showing smaller gray matter volumes in the right supplementary motor area (SMA) of participants with hyperacusis compared to those without hyperacusis. The analysis accounted for total intracranial volume (TIV) as a confounding variable. One single cluster (244 voxels) of gray matter volumes difference was found in the entire brain, shown in cold color map (peak MNI *X*, *Y*, *Z* - coordinates = 9, –8, 69, T = 5.2, P_{FWE} = 0.012 at the voxel-level).

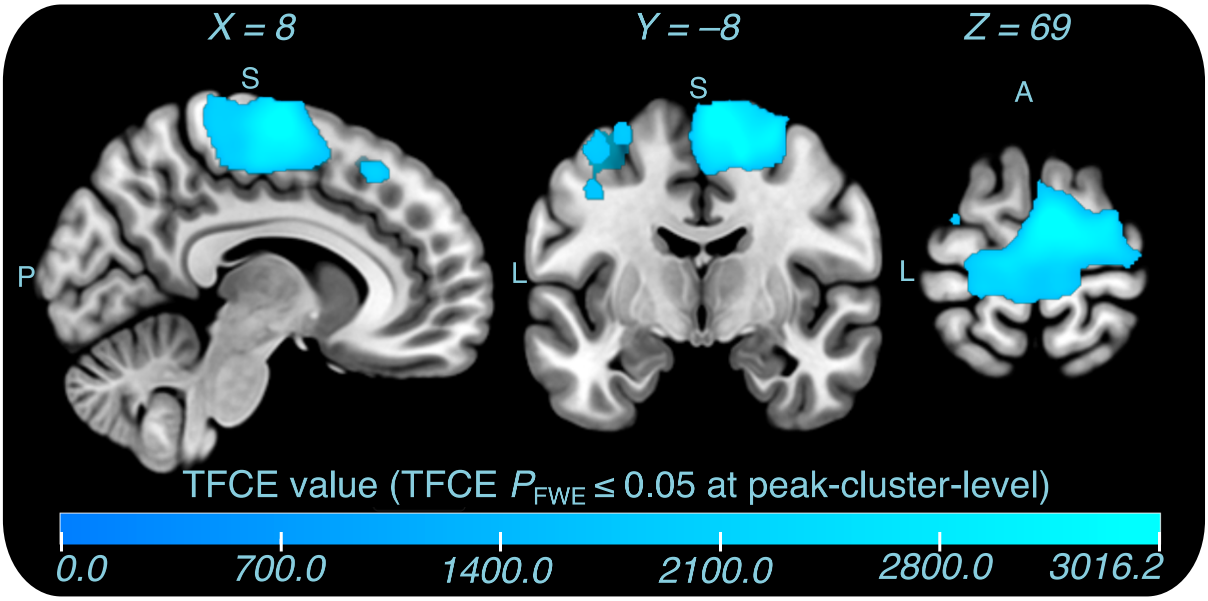

Supplementary Figure S2. Results of the whole-brain between groups comparison showing smaller gray matter volumes in the right supplementary motor area (SMA) of participants with hyperacusis compared to those without hyperacusis. The analysis accounted for age, handedness score, hearing thresholds (PTA 0.25 - 8 kHz for both ears), and total intracranial volume (TIV) as confounding variables. One single cluster (14,276 voxels) of gray matter volumes difference was found in the entire brain (shown in cold color map), however, cluster was extended into the SMA, pre-supplementary motor area, and precentral gyrus bilaterally (peak MNI *X*, *Y*, *Z* - coordinates = 8, –8, 69, TFCE = 3016.2, TFCE P_{FWE} = 0.001 at the peak-cluster-level, permutation = 5000).

Correlation between the right SMA gray matter volumes and scores on the Hyperacusis Questionnaire

Regression analyses were conducted in order to test whether using the Hyperacusis Questionnaire (HQ) scores as a continuous variable (instead of relying on a cut-off score) would produce different results, and to test whether a subset of items from the HQ drove the correlation.

Methods

1. A General Linear Model (GLM) was built with the HQ scores as a covariate of interest, also controlling for the effects of age, handedness, hearing thresholds, and total intracranial volume (TIV). The analysis was performed in SPM12/CAT12.7 in the whole brain with two different statistical thresholds: FWE correction at the voxel level ($P \leq 0.05$), and FWE correction at the cluster level ($P \leq 0.05$, cluster forming threshold $P \leq 0.001$).
2. Gray matter volumes extracted from the right SMA HMAT VOI (see main text) were correlated with the scores on the different subscales of the HQ. Two sets of correlation analyses were conducted: (a) taking into account the original classification into three principal components[1], and (b) using a newer decomposition[2] which proposed a two factors structure based on 10 of the 14 original questions.

Results

1. There was no significant correlation between hyperacusis scores and whole-brain gray matter volumes at the strictest threshold ($P_{FWE} \leq 0.05$). However, relaxing the threshold to $P \leq 0.001$ (with correction at the cluster size level, $P_{FWE} \leq 0.05$) showed a negative

correlation between hyperacusis scores and gray matter volumes with main peaks in the right SMA extending into the left precentral gyrus (Supplementary Table S4).

2 Regressing the right SMA gray matter volumes on the different subscales of the HQ showed significant negative correlations with every subscale of the questionnaire (Supplementary Table S5). The stronger correlations were found for the attentional subscale independently of the manner of grouping the items.

Supplementary Table S4. Results of the regression analysis between hyperacusis scores and whole-brain gray matter volumes including age, handedness scores, hearing thresholds, and total intracranial volume (TIV) as covariates ($P_{FWE} \leq 0.05$ at the cluster-level, cluster forming threshold $P \leq 0.001$, height threshold T = 3.2).

Area	P_{FWE} cluster-level	P uncorrected	MNI coordinates *X*	*Y*	*Z*	Cluster size *k*	T-value	Z-value
	**Negative effect of Hyperacusis**							
SMA (right)	< 0.001	< 0.001	6	−15	52	5326	4.1	3.9
		< 0.001	3	−14	68		4.1	3.9
PreCG (left)		< 0.001	−18	−33	74		4.3	4.0
	**Positive effect of Hyperacusis**							
NS	-	-	-	-	-	-	-	-

NS not significant. PreCG precentral gyrus.

Supplementary Table S5. Person's correlation coefficients between the different subscales of the HQ and the right SMA gray matter volumes (HMAT VOI).

	HQ-attentional	HQ-social	HQ-emotional
	**Khalfa et al.**[1]		
**SMA (right)**	*r* = –0.51<br>*P* < 0.001<br>*n* = 59	*r* = –0.33<br>*P* = 0.011<br>*n* = 59	*r* = –0.34<br>*P* = 0.009<br>*n* = 59
	**Fackrell et al.**[2]		
**SMA (right)**	*r* = –0.44<br>*P* < 0.001<br>*n* = 59	*r* = –0.26<br>*P* = 0.049<br>*n* = 59	-<br>-<br>-

References

1. Khalfa S, Dubal S, Veuillet E, Perez-Diaz F, Jouvent R, Collet L. Psychometric normalization of a hyperacusis questionnaire. *ORL J Otorhinolaryngol Relat Spec*. 2002;64(6):436-442. doi:10.1159/000067570

2. Fackrell K, Fearnley C, Hoare DJ, Sereda M. Hyperacusis Questionnaire as a Tool for Measuring Hypersensitivity to Sound in a Tinnitus Research Population. *Biomed Res Int*. 2015;2015:290425. doi:10.1155/2015/290425
